# Supplementary material for: Spectroscopic Evidence for Photooxidation of Tocopherols in n-Hexane
Source: Molecules. 2021 Jan 22;26(3):571. doi: 10.3390/molecules26030571 (PMC7865854; doi:10.3390/molecules26030571)
Supplement: Supplementary file 1 [file molecules-26-00571-s001.pdf]

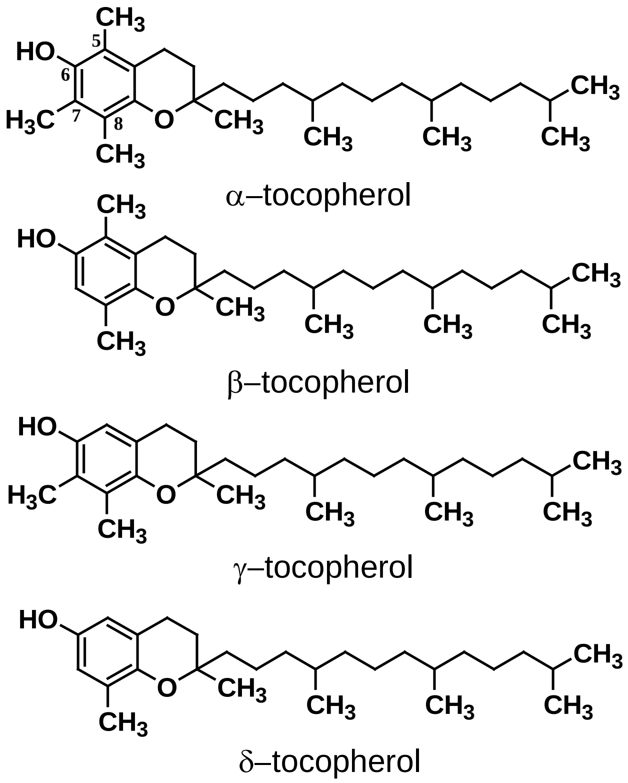

Chart S1. Molecular structure of Tocopherols

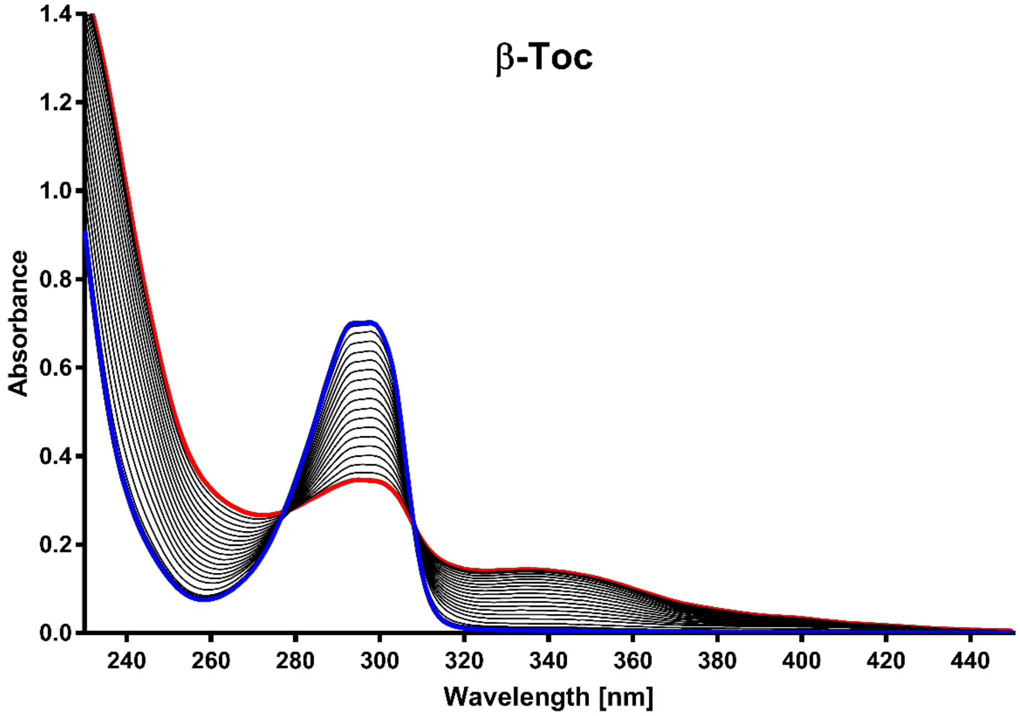

Figure S1. Time-course of absorption spectra of β-Toc. Blue - initial spectrum, red – final spectrum. Irradiation time 509 min. Optical path – 1 cm.

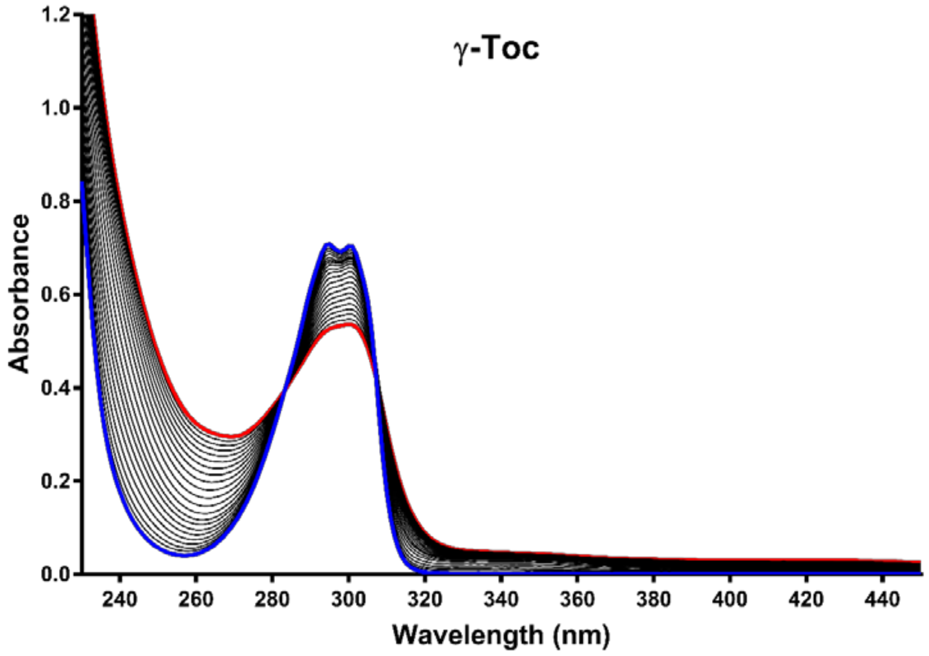

Figure S2. Time-course of absorption spectra of γ-Toc. Blue - initial spectrum, red – final spectrum. Irradiation time 647 min. Optical path – 1 cm.

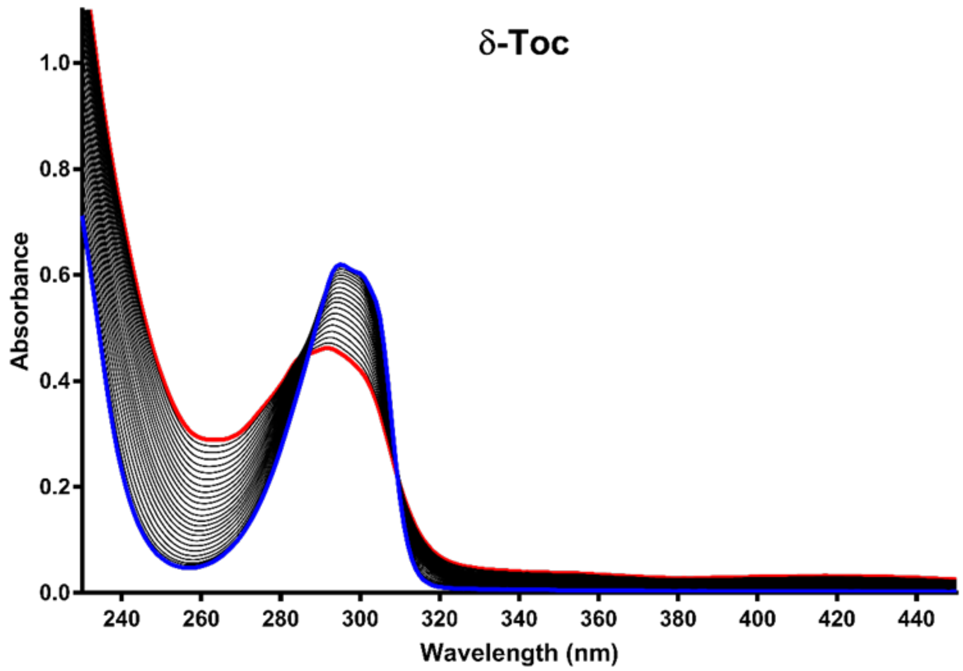

Figure S3. Time-course of absorption spectra of δ-Toc. Blue - initial spectrum, red – final spectrum. Irradiation time 905 min. Optical path – 1 cm.

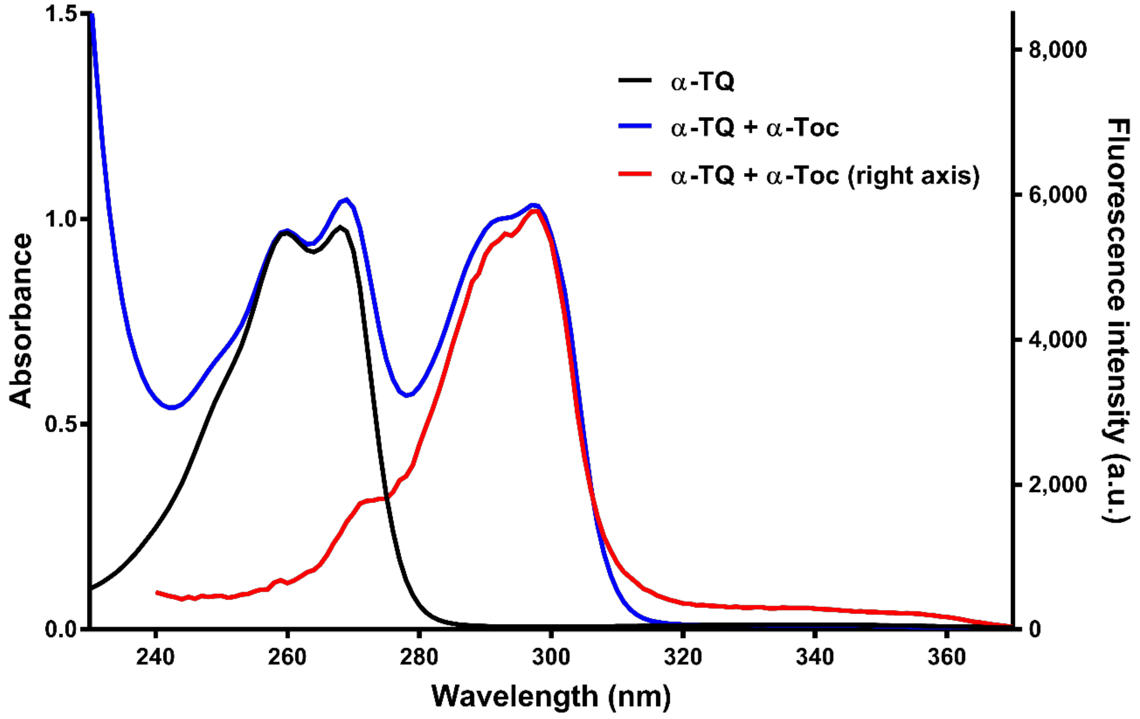

Figure S4. Absorption and excitation spectra of α-Toc with and without α-TQ. Black line – absorption spectrum of α-TQ, blue line – absorption spectrum of α-Toc + α-TQ, red line – excitation spectrum of α-Toc + α-TQ. Samples were non-irradiated. λ_{obs} = 325 nm, optical path - 1 cm.

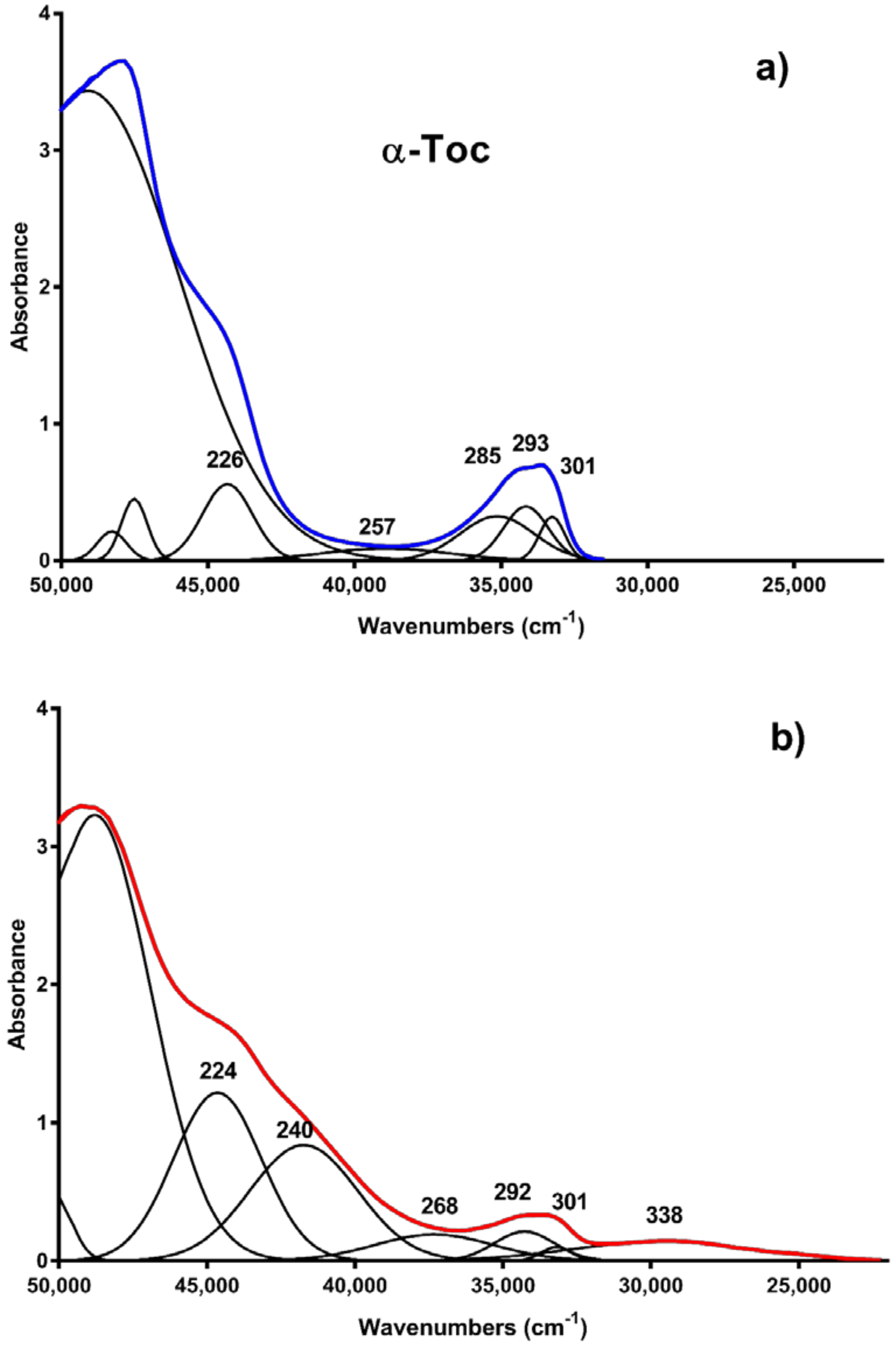

Figure S5. Absorption spectra of α-Toc: a) initial spectrum (R^2 = 0.999981), b) final spectrum of the same sample (R^2 = 0.999978). The numbers on the graph indicate wavelengths in nm. Black lines – Gauss bands after deconvolution.

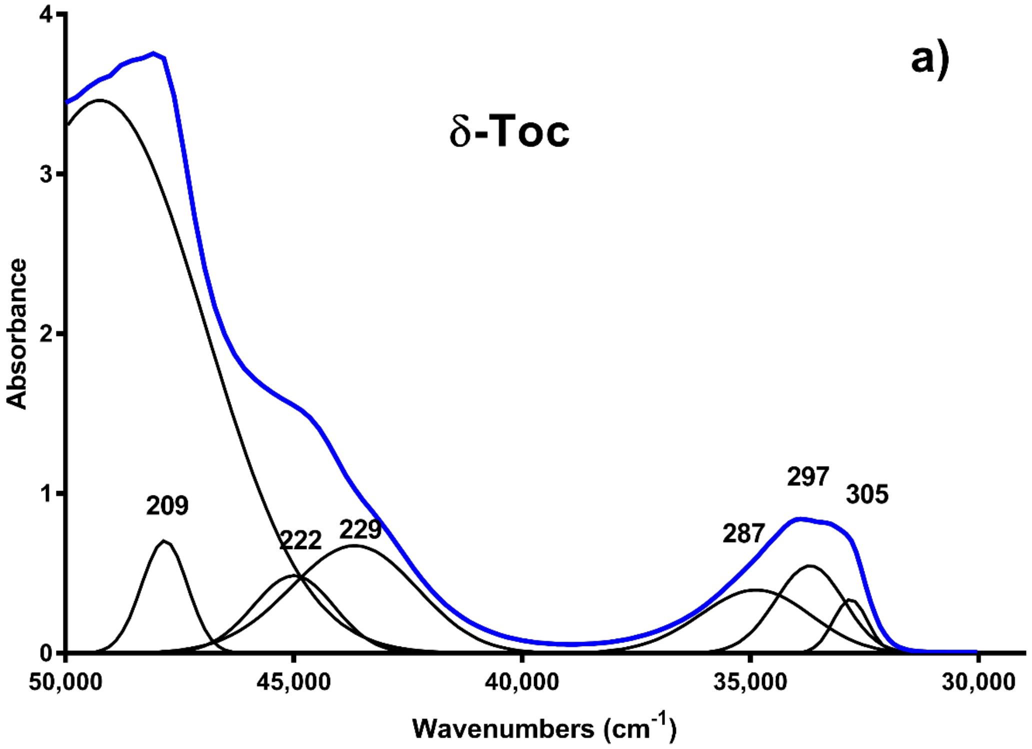

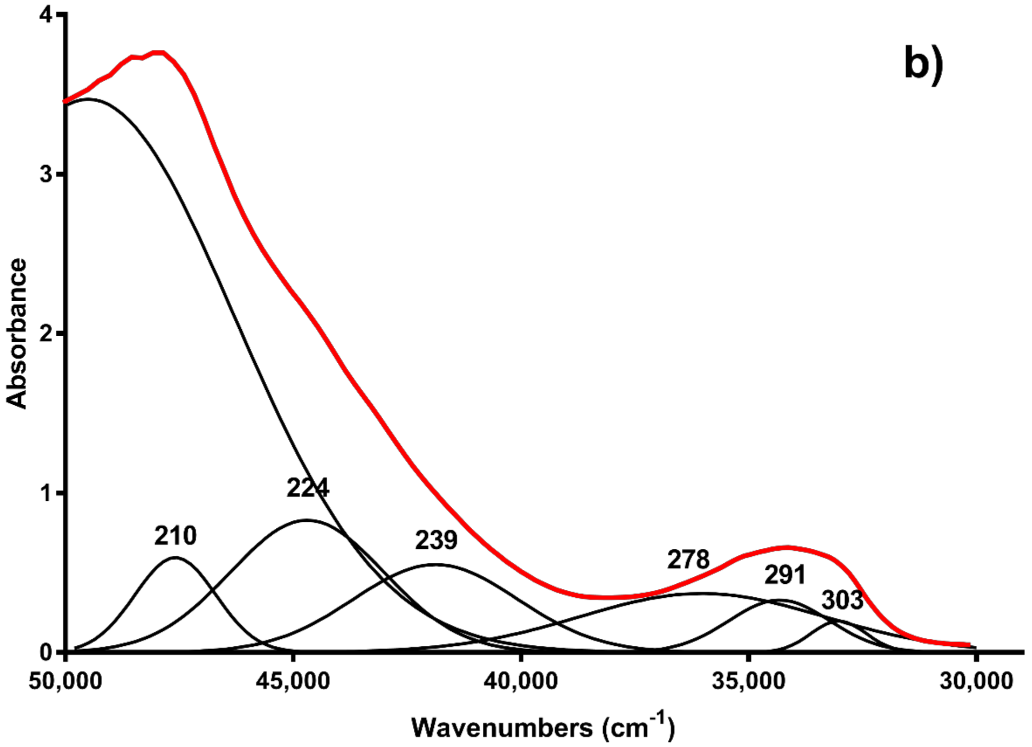

Figure S6. Absorption spectra of δ-Toc: a) initial spectrum (R^2 = 0.999910), b) final spectrum of the same sample (R^2 = 0.999974). The numbers on the graph indicate wavelengths in nm. Black lines – Gauss bands after deconvolution.

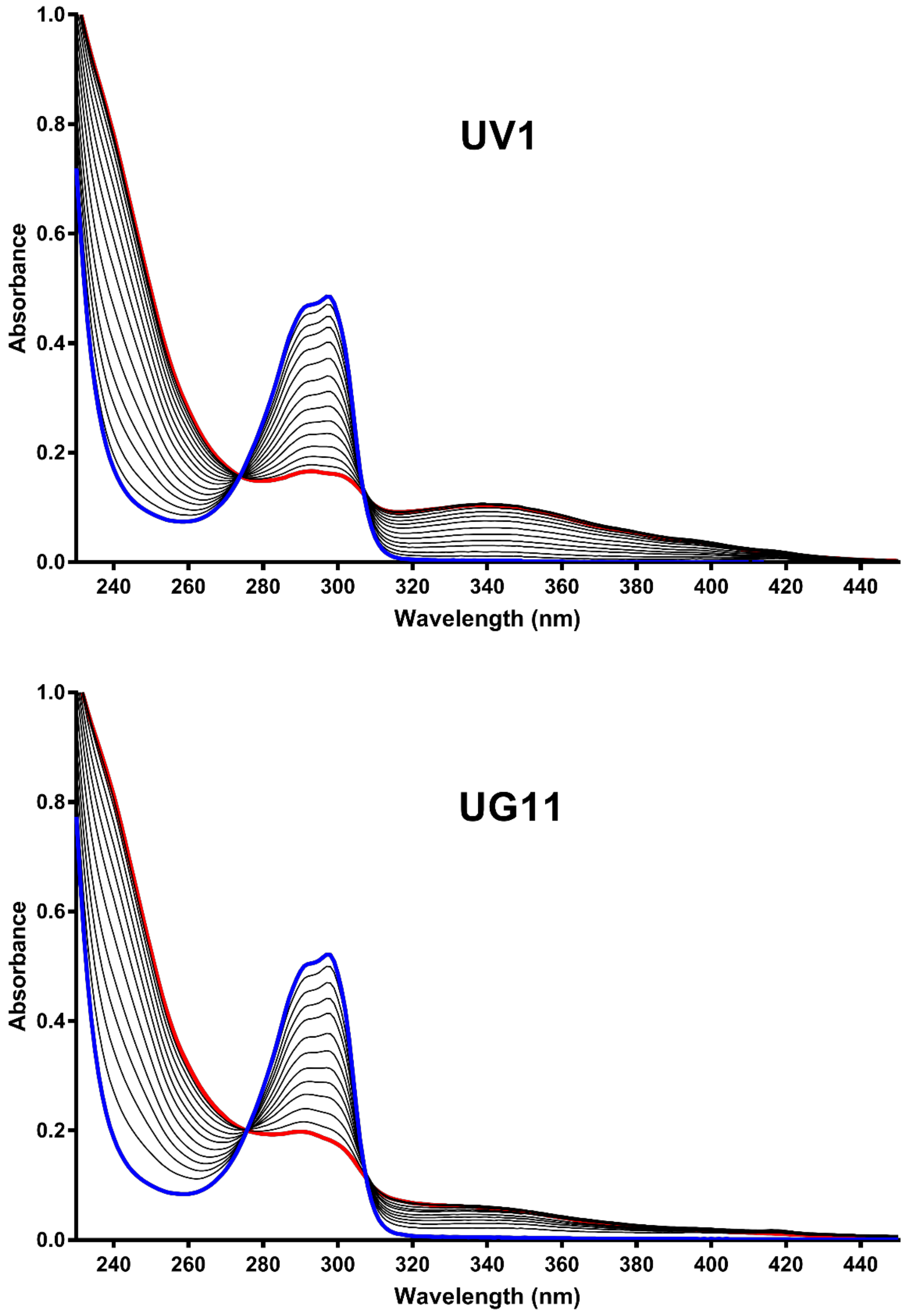

Figure S7. Time-course of spectra of α-Toc irradiated through different UV filters. UV1 – 409 min, UG11 – 83 min. Optical path – 1 cm.

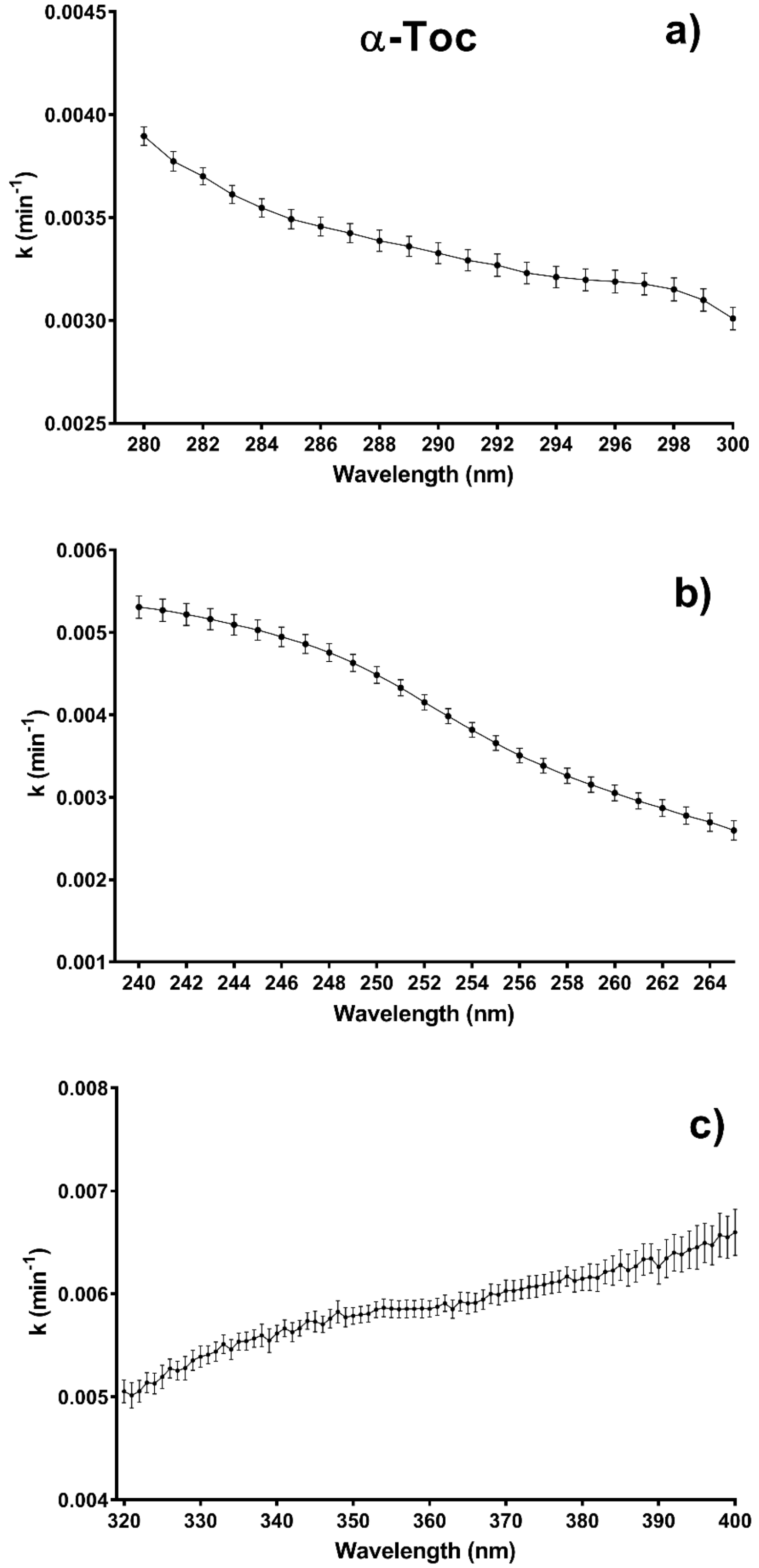

Figure S8. Wavelength dependence of calculated photooxidation rate constants of α-Toc.
Error bars = SD – standard deviation.

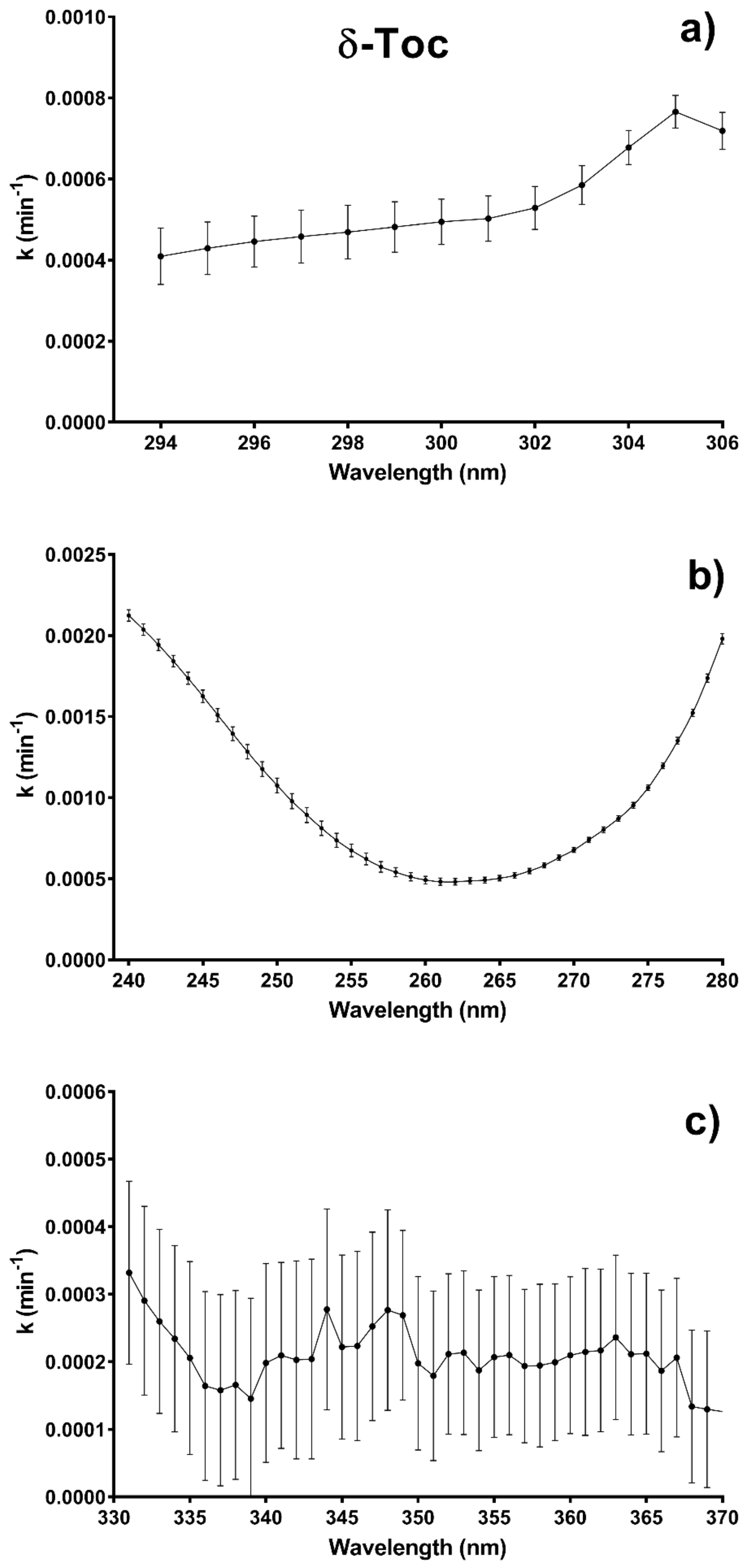

Figure S9. Wavelength dependence of calculated photooxidation rate constants of δ-Toc. Error bars = SD – standard deviation.

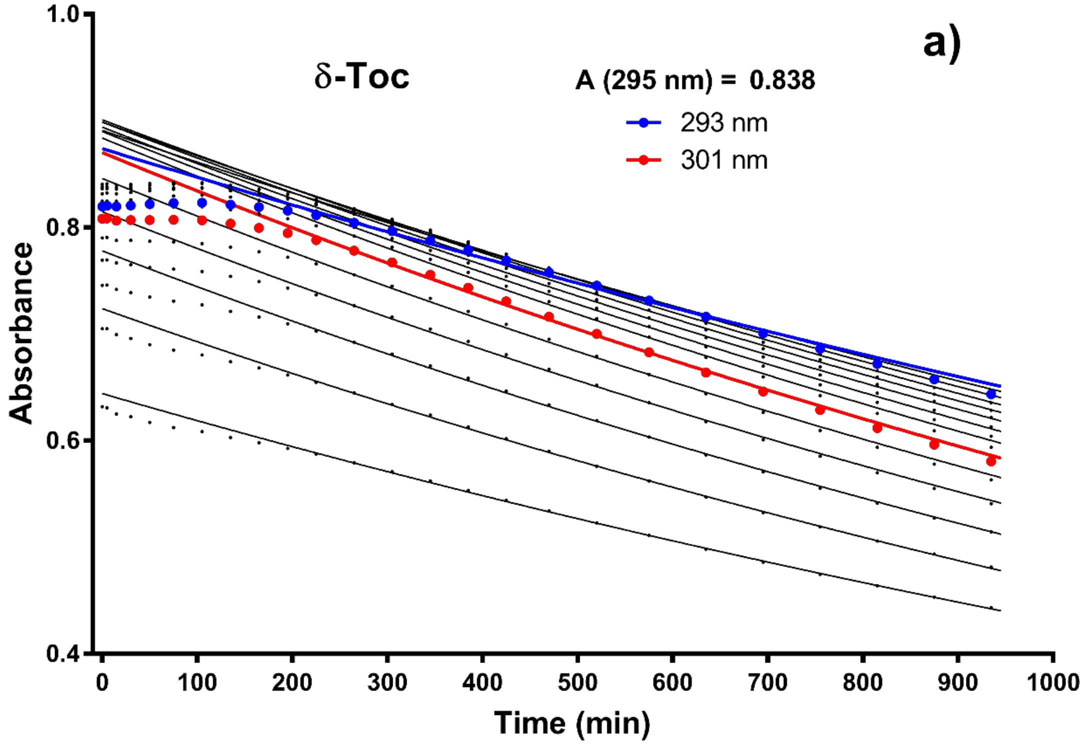

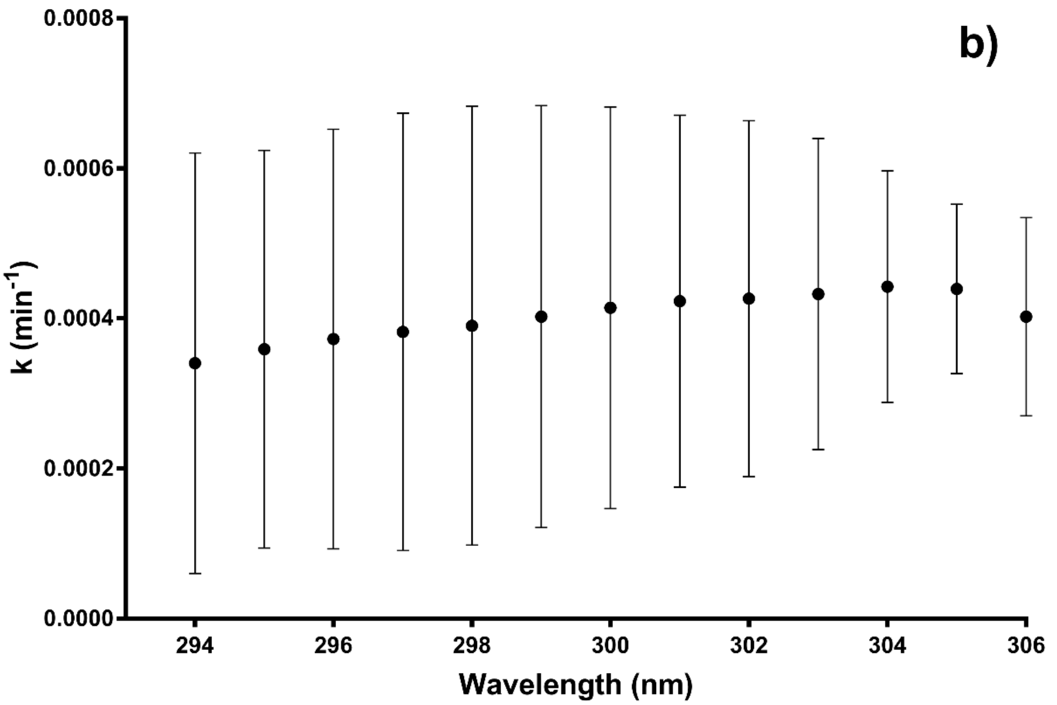

Figure S10. a) Time-course of absorbance of δ-Toc in the range from 294 to 306 nm, b) values of the rate constants in above range. Bars = SD. Sample was non-argonated. Black dots – data obtained from an absorbance. In Fig. a) black lines and dots are for data outside “red and blue” range.

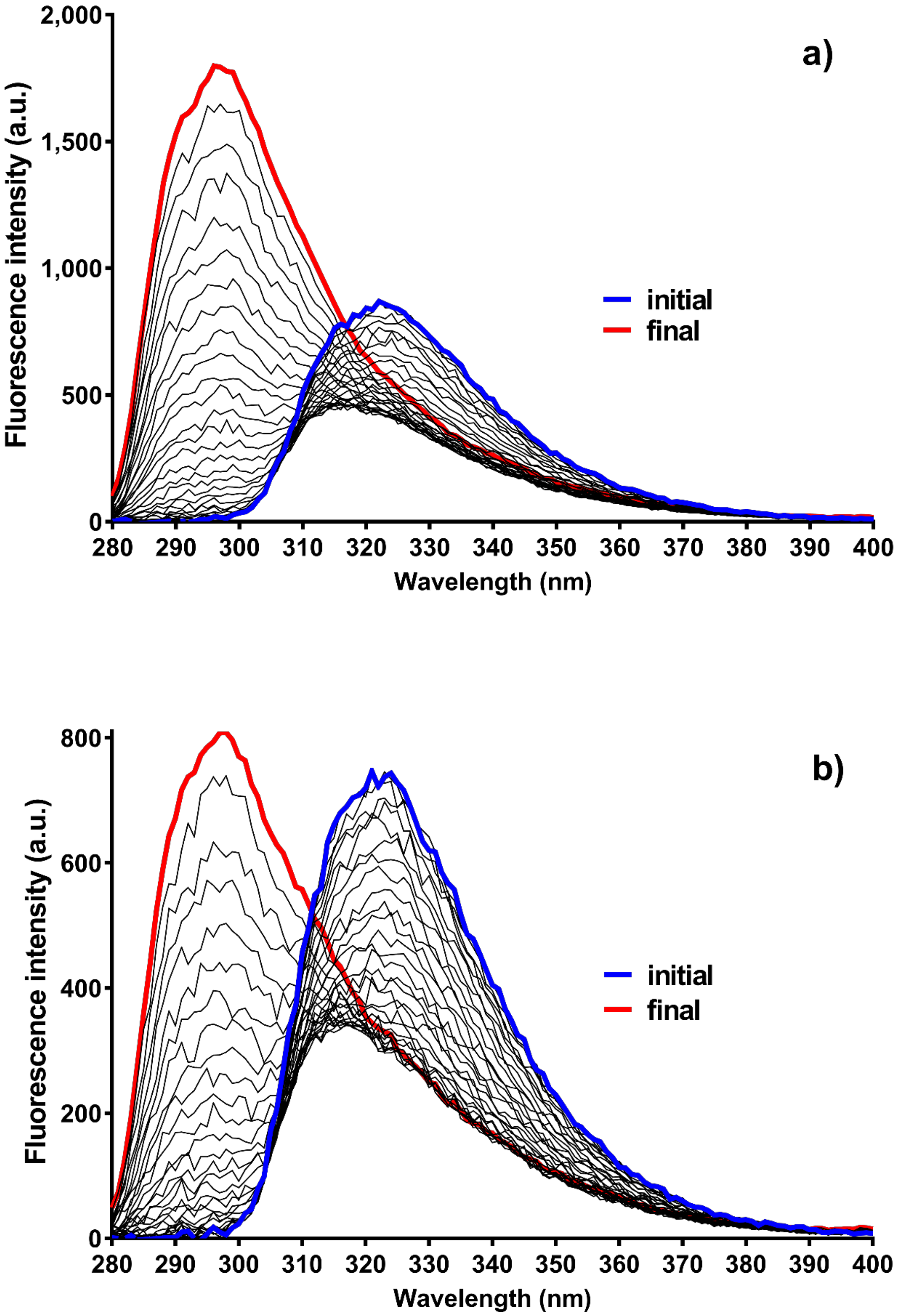

Figure S11. Time-course of fluorescence spectra of argonated a) and non-argonated b) sample of δ-Toc. λ_{exc} = 265 nm. Optical path – 1 cm.

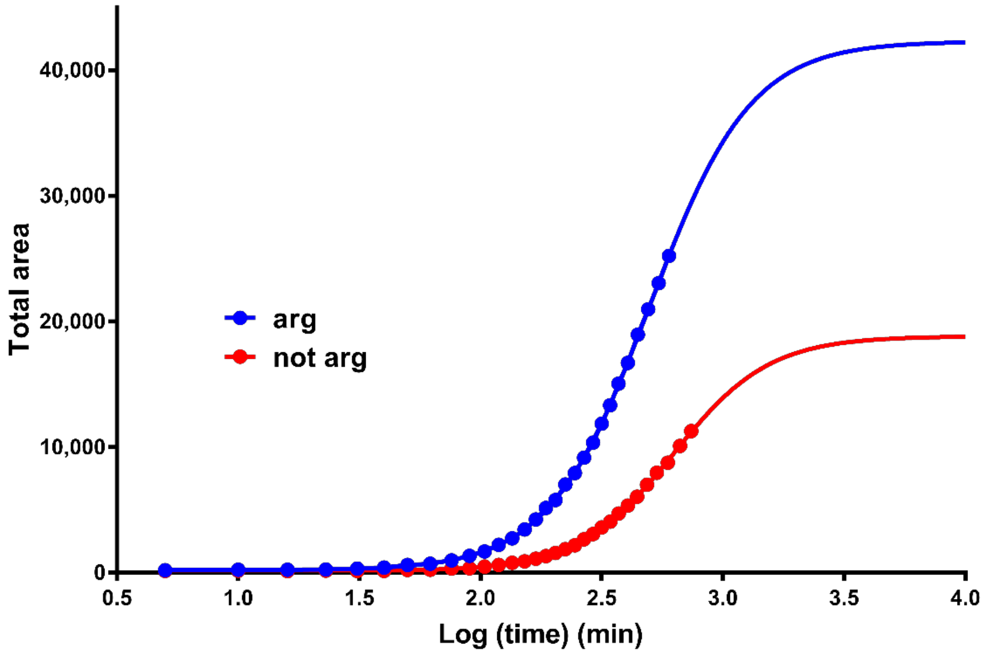

Figure S12. Changes of total area of emission spectra of δ-Toc in the range from 280 to 300 nm. λ_{exc} = 265 nm. Argonated sample time irradiation – 599 min, non-argonated – 740 min.

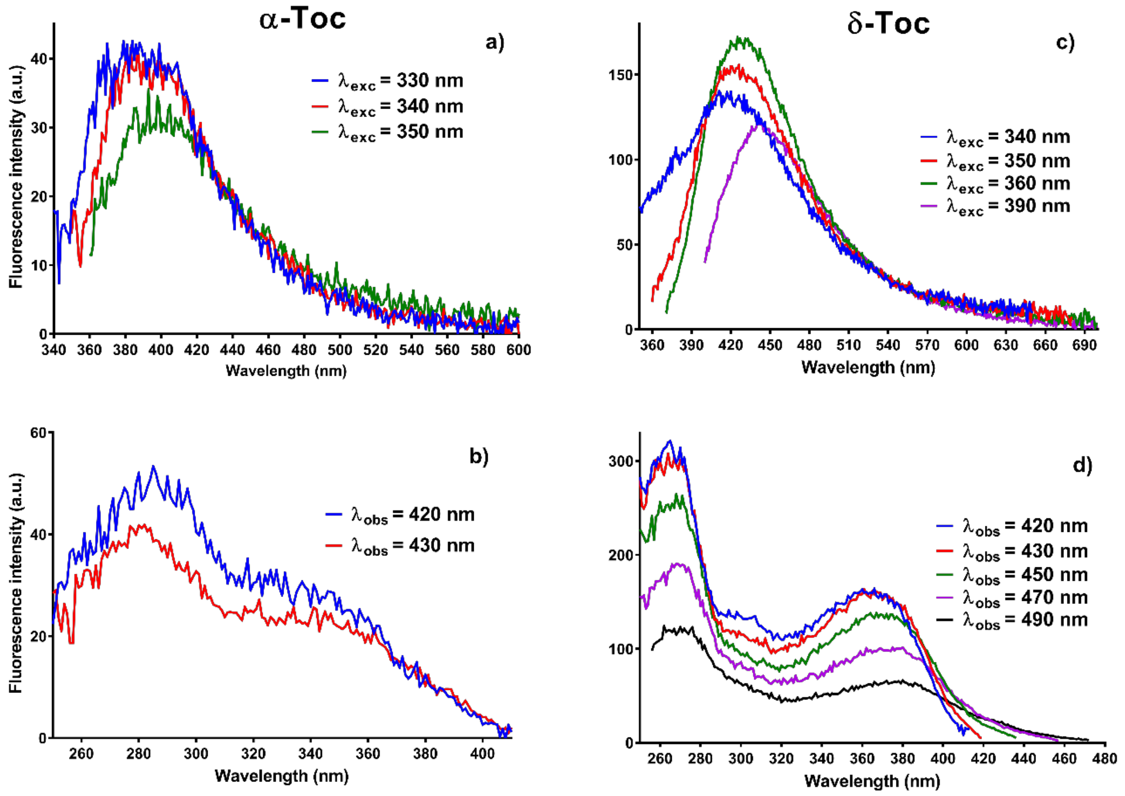

Figure S13. Emission spectra of C-T complexes after sample irradiation of α-Toc - a), δ-Toc – c), and excitation spectra of α-Toc - b), δ-Toc – d). Optical path – 1 cm.

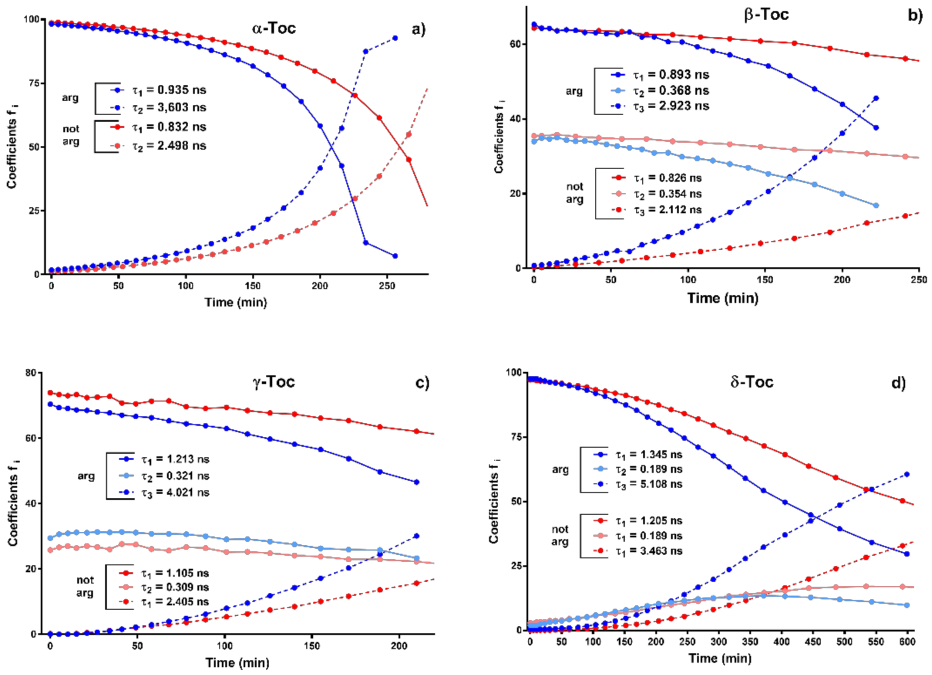

Figure S14. Time-course of Tocs f_i coefficients obtained from Global method. Decay times were measured using λ_{exc} = 283 nm and λ_{obs} = 325 nm.

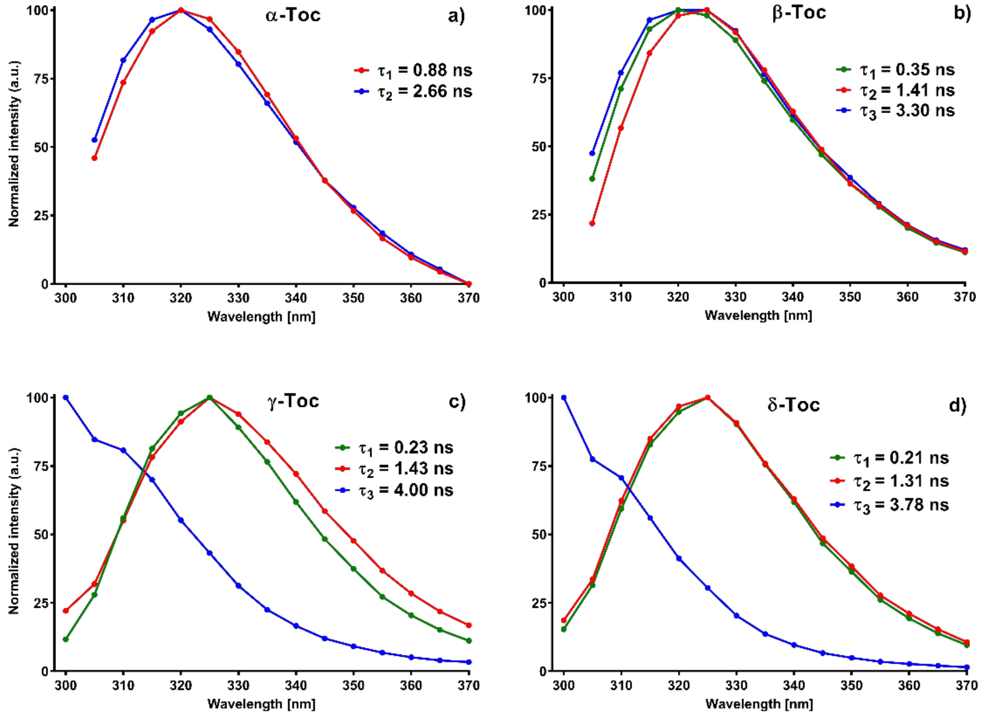

Figure S15. DAS Spectra calculated for final sample of each Toc using TRES method. Decay time were measured using λ_{exc} = 283 nm and observation was from 300 to 370 nm with step = 5 nm.
